# Salivary trimethylamine N-oxide: a novel non-invasive marker for periodontal inflammation

**DOI:** 10.1007/s00784-025-06295-4

**Published:** 2025-03-25

**Authors:** Ceyhan C. Serdar, Zeliha Guney, Nur Balci, Sema M. Altingoz, Muhittin A. Serdar, Sivge Kurgan

**Affiliations:** 1 Department of Medical Biology and Genetics, School of Medicine, Ankara Medipol University, Ankara, Turkey; 2 Department of Periodontology, Faculty of Dentistry, Ankara Medipol University, Ankara, Turkey; 3 Department of Periodontology, Faculty of Dentistry, Istanbul Medipol University, Istanbul, Turkey; 4 Faculty of Dentistry, Department of Periodontology, Lokman Hekim University, Ankara, Turkey; 5 Department of Medical Biochemistry, School of Medicine, Acibadem University, İstanbul, Turkey; 6 Department of Periodontology, Faculty of Dentistry, School of Dentistry, Ankara University, 06500 Cankaya Ankara, Turkey

**Keywords:** Periodontitis, Trimethylamine N-oxide, Liquid biopsy-based diagnostics

## Abstract

**Objectives:**

Trimethylamine N-oxide (TMAO) has been implicated in systemic inflammatory pathways, emphasizing its potential as a biomarker. Elevated plasma TMAO levels have been associated with increased oxidative stress, leading to higher plasma concentrations of TNF-α, a key pro-inflammatory cytokine. Given this systemic inflammatory linkage, saliva—a non-invasive diagnostic medium—offers a unique opportunity to reflect both local and systemic inflammatory changes. This study aimed to evaluate the alterations in salivary and serum TMAO levels in periodontitis and assess the diagnostic potential of salivary TMAO as an indicator of periodontal inflammation.

**Materials and methods:**

Twenty-four patients with periodontitis (Stage III Grade B) and 24 healthy controls were included. Clinical parameters (probing depth (PD), plaque index (PI), bleeding on probing (BOP), and clinical attachment loss (CAL)) were recorded. TMAO levels in saliva and serum were measured using liquid chromatography-mass spectrometry (LC–MS/MS), and TNF-α levels were assessed using Enzyme-Linked ImmunoSorbent Assay (ELISA).

**Results:**

Salivary and serum TMAO levels and salivary TNF-α levels were significantly higher in the periodontitis group (*p* = 0.003, *p* = 0.004, and *p* = 0.031, respectively). Salivary TMAO showed positive correlations with periodontal parameters (*p* < 0.05) and salivary TNF-α levels. A significant positive correlation was also observed between salivary and serum TMAO levels (*p* < 0.001). Salivary TMAO was the accurate biomarker in differentiating between periodontitis and controls (sensitivity = 0.583, specificity = 0.833, AUC = 0.747).

**Conclusions:**

Salivary TMAO demonstrates potential as a non-invasive marker for periodontitis, showing correlations with clinical parameters and inflammatory markers. These findings suggest that TMAO may reflect both local and systemic inflammatory states associated with periodontal disease.

**Clinical relevance:**

Salivary TMAO may serve as a potential non-invasive indicator of periodontitis, as it reflects aspects of both local and systemic inflammation, offering insights into periodontal disease status.

## Introduction

Trimethylamine N-oxide (TMAO), a gut flora-derived metabolite from dietary choline, has emerged as an indicator of atherosclerosis [1]. Circulatory TMAO has been associated with increased cardiovascular risk by disrupting enterohepatic cholesterol and bile acid metabolism, upregulating macrophage scavenger receptor expression, and promoting endothelial dysfunction, oxidative stress, and inflammation [2–4]. It activates nuclear factor kappa B (NF-κB), leading to the elevated expression of pro-inflammatory cytokines such as tumor necrosis factor-alpha (TNF-α), interleukin-6 (IL-6), and IL-1β [5] through IL-1-related pro-inflammatory pathways [2, 3]. Simultaneously, TMAO suppresses anti-inflammatory cytokines like IL-10 [6], further contributing to systemic inflammation and atherosclerosis progression. Additionally, TMAO can contribute to inflammatory processes by enhancing macrophage chemotaxis and increasing the expression of inflammatory cytokines like TNF-α [7, 8], which plays a crucial role in immune responses and inflammation and has been demonstrated as a crucial participant also during the development of periodontal diseases [9]. Interestingly, TMAO has been detected in cerebrospinal fluid [10] and has been implicated in neurodegenerative diseases, including Alzheimer’s [11] and Parkinson’s [12], through its role in amyloid-beta aggregation and tau protein stabilization [13]. A clinical study has highlighted elevated circulating TMAO levels in periodontitis, correlating with *in-vitro* endothelial dysfunction [3]. Additionally, TMAO’s association with oral pathogens, such as *Porphyromonas gingivalis* and *Aggregatibacter actinomycetemcomitans* in myocardial infarction patients, underscores its potential link to oral dysbiosis [14]. However, the precise role of TMAO in the local (salivary) versus systemic (serum) levels associated with periodontitis remains poorly defined.

According to the literature, in both in vitro and in vivo studies, elevated TMAO levels have been linked to periodontal disease and its systemic effects [3, 8, 15–17]. In vivo studies in mice demonstrate that periodontitis-induced gut dysbiosis increases plasma TMAO [16], exacerbating atherosclerosis [17], endothelial dysfunction, and systemic inflammation via IL-6 and FMO3 upregulation [8, 15]. In vitro studies confirm that TMAO impairs endothelial progenitor cell function, induces pyroptosis, and promotes inflammatory responses [3], suggesting a mechanistic link between periodontitis, TMAO metabolism, and cardiovascular risk. While studies have explored the interplay between periodontitis and cardiovascular risk via circulating TMAO levels, limited attention has been given to salivary TMAO levels to evaluate its contribution to periodontal inflammation [3, 14]. Unlike serum TMAO, salivary TMAO offers a novel, non-invasive diagnostic approach that can be integrated into routine clinical evaluations. This perspective underscores the unique value of salivary biomarkers, particularly for localized inflammatory conditions like periodontitis, and highlights their potential in bridging oral and systemic health diagnostics. This study aims to evaluate the independent effects of periodontitis on TMAO and TNF-α levels in saliva and serum. By focusing on salivary TMAO, the study provides novel insights into its potential as a localized biomarker and highlights its diagnostic utility in non-invasive clinical applications, advancing our understanding of its link to cardiovascular disease pathways.

## Materials and methods

### Study population and clinical examination

The study’s design and implementation received approval from Istanbul Medipol University Human Research Ethics Committee (date: 07.12.2023; Number: 1004). The research adhered to the principles outlined in the Declaration of Helsinki, as revised in 2013. The recruitment was between December 2023 and April 2024 in Istanbul Medipol University of Dentistry, Department of Periodontology. Before participation, all individuals who agreed to be part of the study provided formal informed consent by signing a consent form.

Inclusion criteria of the study were (1) being over 18 and under 65 years of age; (2) having at least 20 natural teeth excluding the third molars; and (3) being systematically healthy. Exclusion criteria of the study were (1) being smoker; (2) chronic use of any systemic medication (3) use of antibiotics and/or anti-inflammatory steroids, nonsteroidal anti-inflammatory drugs, immunosuppressants, beta-blockers, calcium channel blockers, anticoagulants, or hormonal contraceptives within 3 months preceding the study; (4) having periodontal treatment (previous 6 months); (5) pregnancy or lactation; and (6) use of orthodontic appliances (7) DMFT index (D = decay, M = missing, F = filling; T per tooth) > 2 [18].

**Fig. 1:**
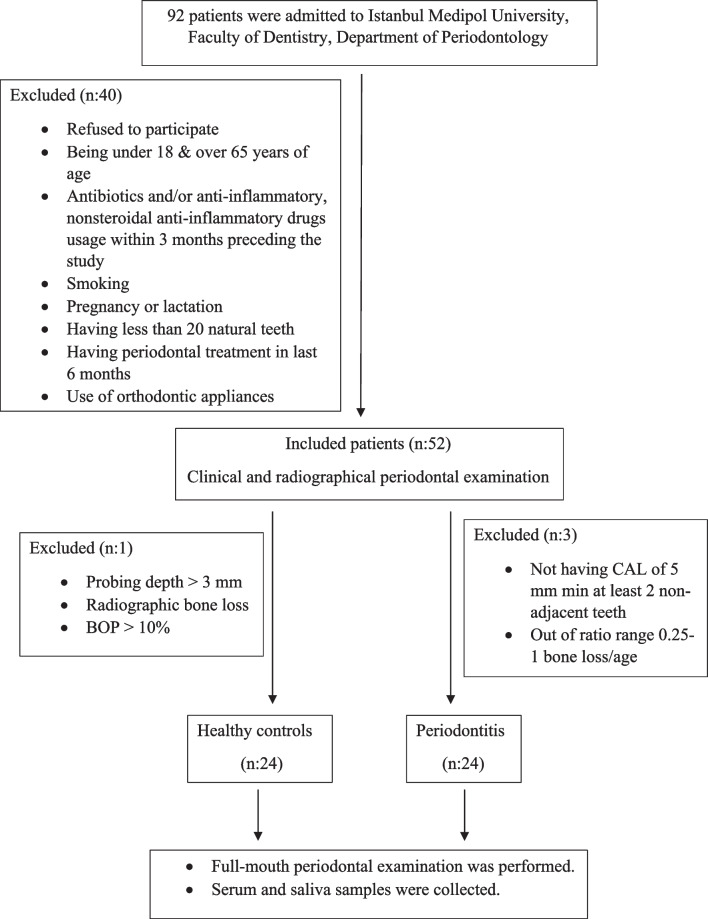
The STROBE Flow chart of the study

A comprehensive periodontal evaluation was conducted, which included radiographic imaging (orthopantomographs) and clinical assessment with periodontal probe.^1^ All clinical parameters were recorded by a sole examiner (NB). Recorded parameters were probing depth (PD), plaque index (PI), bleeding on probing (BOP), and clinical attachment loss (CAL). The clinical diagnosis of patients’ periodontal status was categorized according to the 2017 Classification of Periodontal and Peri-Implant Diseases and Conditions [19]. Control group individuals had no periodontal disease history or symptoms with clinically healthy periodontium (BOP < 10%, PD ≤ 3 mm) and lacked clinical signs of gingival inflammation with well-maintained oral hygiene. The periodontitis group had an interdental CAL detectable at ≥ 2 non‐adjacent teeth or buccal or oral CAL ≥ 3 mm with pocket depth > 3 mm detectable at ≥ 2 teeth. The following criteria were used to define stage III: (1) Radiographic alveolar bone loss in the middle or apical third of the root. (2) Tooth loss due to periodontitis ≤ 4. (3) Interdental CAL in the highest lost area ≥ 5 mm. The additional condition for grade B was % bone loss/age 0.25–1. The study included 92 patients who applied to the Department of Periodontology at Istanbul Medipol University, Faculty of Dentistry. Two of the 92 patients declined to participate in the trial. Fourteen patients were eliminated because they had recently taken antibiotics, five patients were discarded because they had recently received periodontal therapy, and nineteen patients were excluded because they had systemic diseases, resulting in a final sample size of twenty-four patients with periodontitis and twenty-four periodontally healthy control subjects as presented in the STROBE checklist (Fig. 1).

### Saliva and serum samples

Unstimulated saliva was collected from both periodontitis patients and healthy individuals after the protocol was explained in detail. At first, participants were instructed to rinse their mouths using filtered water and seated calmly while they spit into a disposable tube for 10 min, 5 min later rinsing [20]. Saliva samples were prepared for storage by centrifugation at 3000 × g for 10 min [21].

Standard venipunctures were used to collect blood; the samples were stored at room temperature for 30 min. Serum was prepared from blood samples by centrifugation at 4000 × g for 10 min. Until the analysis date, all serum and saliva samples have been transferred into Eppendorf tubes and preserved at −80^o^ C [22].

### Determination of salivary and serum TNF-α levels

Salivary and serum levels of the tumor necrosis factor-alpha (TNF-α) were measured using a commercial ELISA kit^2^ according to instructions provided by the manufacturer. The standard curve of the assay was used to determine the analyte concentrations in each sample. The assay’s intra- and inter-assay precision values were 9.8% and 8.5%, respectively. The detection range of the commercial kit for TNF-α quantification was specified as 0.31–20.0 pg/mL. All samples were analyzed twice, and the mean values were used for subsequent calculations.

### Determination of salivary and serum TMAO levels

Liquid chromatography-mass spectrometry (LC–MS/MS) was used for the estimation of TMAO levels in saliva and serum, with modifications made to the method outlined by Li et al. [23], Buczko et al. [24]. In our study, 50 uL salivary or serum sample was mixed with 10 uL TMAO-d9 (IS, 500 ng/mL) and 200 uL acetonitrile (ACN), then vortexed for 10 min and then centrifuged (14,000 rpm for 5 min at 4 °C). Then, 50 μL of each supernatant was transferred to a new tube and mixed with 50 μL of 30% ACN solution, and 5 μL from each final mixture was injected into the analysis system. To identify and quantify TMAO, samples were analyzed by an LC–MS/MS^3^ system equipped with a Thermo Dionex Ultimate 3000 UHPLC system and a TSQ Quantum Access Max quadrupole mass spectrometer (USA). The C18 column (100 × 3 mm, 3 μm) purchased in Phenomenex was connected to the LC system for the separation of the analytes. Separate mobile phases were used by two individual pumps. Pump A contained a 5 mM ammonium acetate solution, and pump B held acetonitrile. The gradient elution started with 80% A at 0.0 min, linearly reducing to 20% in 1.5 min and returning to 80% at 2.5 min until 6.0 min. The oven was kept constant at 30 °C, and the flow rate of the mobile phase was 0.5 mL/min. The capillary was set to 225 °C, and the temperature of the vaporizer was 350 °C. The sheat gas pressure was 25 Arb, and the aux gas pressure was 15 Arb. The MRM values (and coalition gas) were as follows: TMAO [76.2 → 58.2 (20 V)], TMAO-d9 [85.3 → 66.2 (20 V)].

### Statistical analysis

The sample size analysis was carried out a priori with special software.^4^ Since there is no similar study in the literature, the effect size was determined as 0.87 as a result of our preliminary study. The sample size with a power of 80% and an alpha value of 5% was calculated to be at least 22 for each group, for a total of 44 people. We decided to include 24 participants in each group to increase the power of the study.

Statistical software^5^ that is available commercially was used for all analyses. The Shapiro–Wilk test was used to evaluate the normality of the data. Mann–Whitney U test was utilized for evaluating normally distributed data, whereas the Kruskal–Wallis test was utilized for evaluating non-normally distributed data. To find relationships between biochemical and periodontal clinical data, the Spearman correlation was used. ROC analysis for differentiation of periodontitis from healthy individuals and area under the curve (AUC) analyses for salivary biomarkers were also performed. The level of significance was set at 0.05.

## Results

### Study population and periodontal clinical parameters

The study included twenty-four patients with periodontitis (P group; *n* = 24; Stage III, Grade B; female = 11, male = 13; mean age: 47.08 ± 8.89 years) and twenty-four periodontally healthy control subjects (C group; *n* = 24; female = 13, male = 11; mean age: 44.5 ± 6.79 years) (Fig. 1; Table 1).

**Table 1:** Demographic, biochemical, and clinical parameters of periodontitis and control groups

Parameters	Control *n* = 24	Periodontitis *n* = 24	*p*
Age (year)*	44.5 ± 6.79	47.08 ± 8.89	0.274
Gender F/M	13/11	11/13	0.564
PI*	0.98 ± 0.33	2.40 ± 0.35	** < 0.001**
PD (mm)*	1.31 ± 0.17	3.48 ± 0.33	** < 0.001**
BOP (%)*	3.54 ± 2.47	70.46 ± 24.56	** < 0.001**
CAL (mm)*	1.31 ± 0.17	3.76 ± 0.52	** < 0.001**

Abbreviations: *PI* plaque index, *PD* probing depth, *BOP* bleeding on probing, *CAL* clinical attachment lost

^*^Data shown as mean ± standard deviation and **median (IQR). Statistical difference with the control group *p* < 0.05

There were no statistically significant differences between the C and P groups in terms of age and sex (*p* = 0.274 and *p* = 0.564, respectively). All measured clinical periodontal parameters (PI, PD, CAL, BOP) were found to be higher in the periodontitis group than the controls. This finding was statistically significant (*p* < 0.001) (Table 1).

**Fig. 2:**
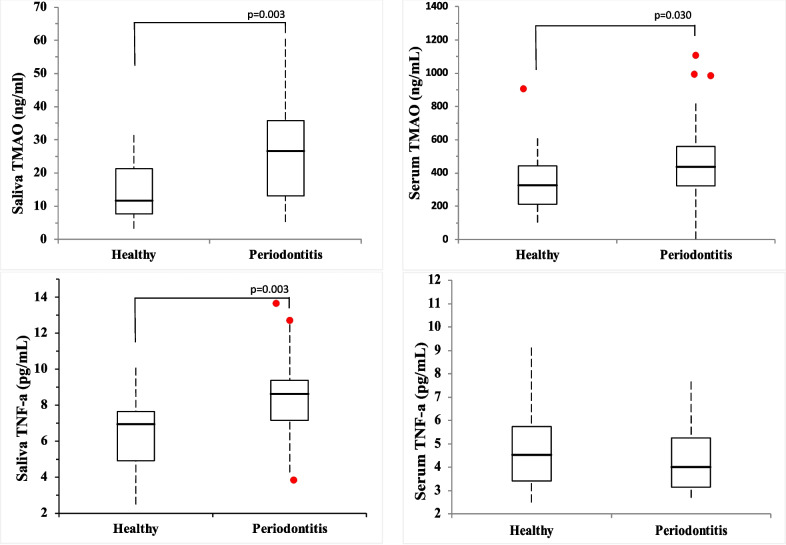
Salivary and serum levels of TMAO and TNFα levels in control (C) and periodontitis (P) groups. Box-and-whisker plots with the median (horizontal line), interquartile range (box), and outlier (circles) values are shown. Significantly different (*p* < 0.05)

Salivary and serum TNF-α and TMAO levels are shown in Table 1 and Fig. 2. The concentrations of TMAO and TNF-α in saliva were higher in the periodontitis group compared to controls (respectively; *p* = 0.003 and *p* = 0.004), and this finding was statistically significant. Likewise, serum levels of TMAO were statistically significantly higher in the periodontitis group than in the controls (*p* = 0.031). There was no statistically significant difference between the periodontitis and control groups in terms of serum TNF-α levels (*p* > 0.05).

### Correlations between biochemical and clinical periodontal parameters

Data from all 48 individuals were used for the correlation analysis (Table 2). Salivary TMAO and TNF-α concentrations were positively and significantly correlated with PD, BOP, and CAL (*p* < 0.05,* p* < 0.001; Table 2). PI was significantly correlated with only salivary TMAO with a low degree of correlation (*r* = 0.361, *p* < 0.05). Age was positively correlated with serum and saliva TMAO levels (*p* < 0.05). Similarly, a positive association was found between saliva TNF-α and serum& saliva TMAO levels (respectively; *r* = 0.307 and 0.290; *p* < 0.05; Table 2). The positive correlation between saliva and serum TMAO was the strongest, with *r* = 0.658 (*p* < 0.001).

**Table 2:** Correlations between biomarkers and periodontal clinical parameters (Spearman correlation coefficients, *r*)

Variables	Salivary TNF-α	Salivary TMAO	Serum TNF-α	Serum TMAO
Salivary TNF-α	-	0,290*	0,144	0,307*
Salivary TMAO	-	-	0,063	0,658**
Serum TNF-α	-	-	**-**	0,072
Age	−0.005	0,332*	−0,083	0,339*
PD (mm)	0.383*	0,412*	0,026	0,261
BOP (%)	0.459**	0,370*	−0,121	0,228
PI	0.186	0,361*	−0,209	0,271
CAL (mm)	0,358*	0,365*	−0,006	0,233

Spearman correlation test. * *p* < 0.05; ** *p* < 0.001

### Receiver operating characteristic (ROC) analysis

**Fig. 3:**
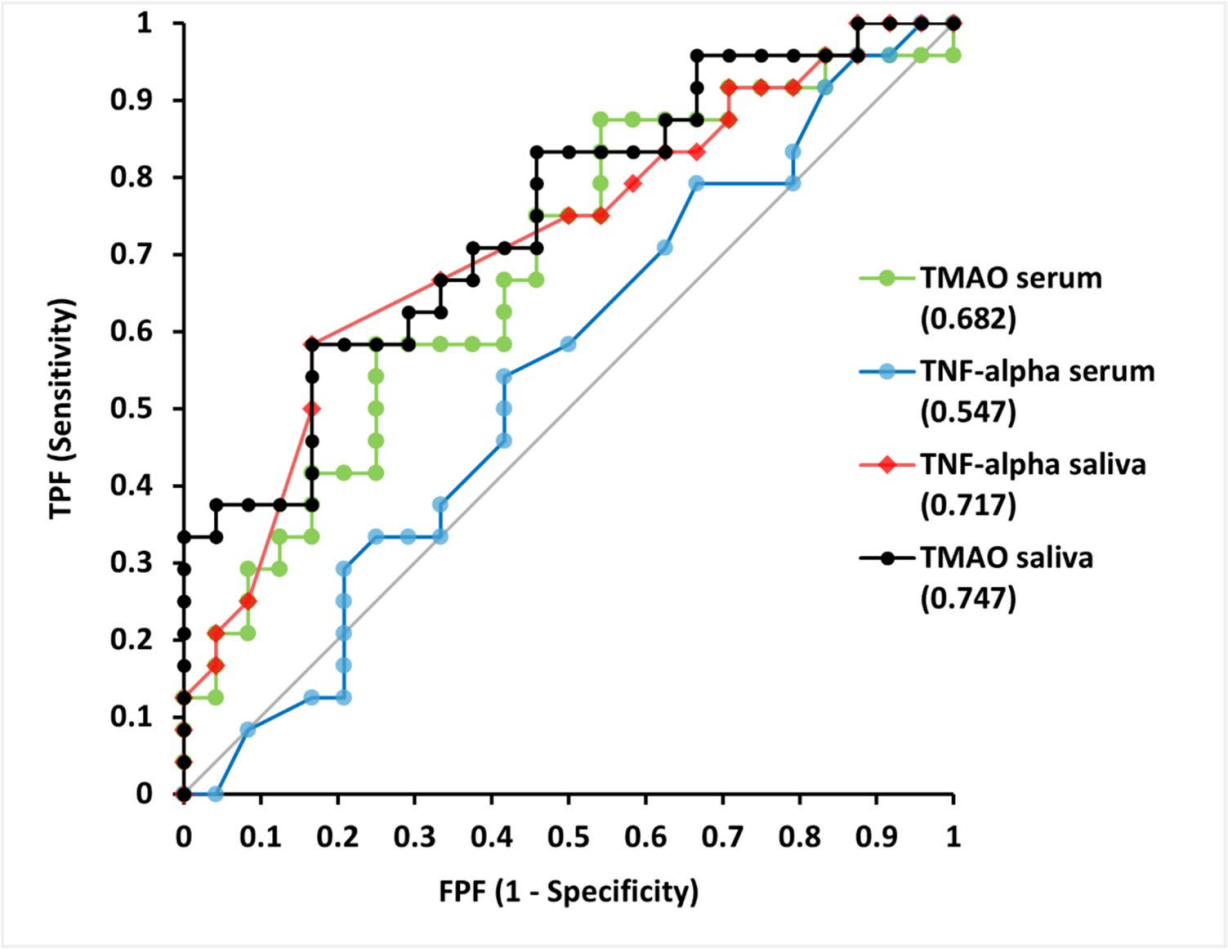
ROC analyses for salivary and serum levels of TMAO and TNF-α. FPF: false predictive fraction; TPF: true predictive fraction

The results of the ROC analyses are shown in Fig. 3. Sensitivity, specificity, and corresponding area under the curve (AUC) values are provided in Table 3. The cut-off point for salivary TMAO, which was identified as the most efficient biomarker of all four parameters for differentiating between periodontitis patients and controls (sensitivity = 0.583, specificity = 0.833, AUC = 0.747), was ​​determined to be 22.67 nmol/L.

**Table 3:** Sensitivity, specificity, and corresponding area under the curve (AUC) values of the ROC analyses for salivary and serum levels of TMAO and TNF-α

Variables	AUC	95% CI	SE	p
Salivary TMAO	0.747	0.608to 0.885	0.0708	**0.0005**
Serum TMAO	0.682	0.529to 0.836	0.0782	**0.0198**
Salivary TNF-α	0.717	0.570to 0.864	0.0748	**0.0060**
Serum TNF-α	0.547	0.380to 0.713	0.0850	0.0581

### Regression analyses

Ordinal regression analyses revealed significant association between higher salivary TNF-α and TMAO levels and higher odds to present periodontitis in an age-adjusted model (OR: 1.490; 95% CI: 1.021, 2.174; *p* = 0.039 for TNF-α and OR: 1.094; 95% CI: 1.003, 1.193; *p* = 0.044 for TMAO) (Table 4).

**Table 4:** Regression analyses

Source	Value	Wald	p	Odds ratio	Lower bound (95%)	Upper bound (95%)
Intercept	−3.566	1.454	0.228			
Salivary TNF-α	0.399	4.275	**0.039**	**1.490**	**1.021**	**2.174**
Salivary TMAO	0.090	4.073	**0.044**	**1.094**	**1.003**	**1.193**
Serum TNF-α	−0.280	1.208	0.272	0.756	0.459	1.245
Serum TMAO	0.000	0.010	0.921	1.000	0.995	1.004
Age	0.005	0.010	0.921	1.005	0.910	1.110

## Discussion

Our study provides new insights into the role of salivary trimethylamine N-oxide (TMAO) as a biomarker for periodontitis, demonstrating elevated salivary and serum TMAO levels in individuals with periodontitis compared to healthy controls. This finding suggests that TMAO is not only a systemic metabolite associated with cardiovascular disease but may also be involved in localized inflammatory responses within the oral cavity. While previous research has primarily focused on circulating TMAO in relation to systemic diseases [3, 14], particularly cardiovascular conditions, our findings highlight its potential relevance within the oral cavity and periodontal inflammatory processes. The significant correlation between salivary TMAO and clinical periodontal parameters, including probing depth (PD), clinical attachment loss (CAL), and bleeding on probing (BOP), suggests that TMAO may be influenced by local periodontal inflammation.

Biomarker-based diagnostic methods provide significant advantages in identifying individuals at risk for disease onset or progression, offering insights beyond traditional clinical parameters such as gingival bleeding, clinical attachment loss, and radiographic bone loss [4, 25]. In periodontology, point-of-care (PoC) tests have facilitated the rapid detection of periodontal diseases by analyzing biomarkers in oral fluids—including saliva, oral rinse, and gingival crevicular fluid—without requiring laboratory-based assessments [26, 27]. Among these, the active-matrix metalloproteinase-8 (aMMP-8) PoC test has been extensively studied and has demonstrated a strong ability to differentiate periodontitis patients from periodontally healthy individuals [28–31]. However, its accuracy may be influenced by systemic conditions, such as Crohn’s disease (CD), where reported sensitivity and specificity values have been lower compared to systemically healthy individuals [32]. While aMMP-8 remains a well-established biomarker for assessing periodontal disease, TMAO has emerged as another potential biomarker that may provide complementary insights, particularly in the context of systemic inflammatory conditions. Unlike aMMP-8, which primarily indicates periodontal tissue degradation, TMAO is associated with broader inflammatory pathways. As a catalytically active and tissue-destructive enzyme, MMP-8 plays a key role in progressive periodontal lesions [26], whereas TMAO has been implicated in endothelial dysfunction, oxidative stress, and inflammatory gene expression [3, 8, 15]. Given that TMAO is recognized for its systemic effects, including modulation of nitric oxide (NO) bioavailability and superoxide-driven oxidative stress [4], its presence in saliva may provide a link between oral and systemic inflammation.

A key finding in our study was that ordinal regression analyses revealed a significant association between higher salivary TNF-α and TMAO levels and increased odds of having periodontitis, even after adjusting for age. This suggests that these biomarkers may serve as independent indicators of periodontal disease risk, beyond the influence of aging-related inflammatory changes. Given that TNF-α is a well-established pro-inflammatory cytokine involved in periodontal destruction [33], its association with TMAO levels and periodontitis status further supports the role of inflammatory-metabolic interactions in periodontal pathogenesis.

One possible explanation for the observed elevation in salivary TMAO is its interaction with oral microbiota. It is well established that TMAO is derived from the microbial metabolism of dietary precursors such as choline, betaine, and carnitine, which are subsequently oxidized in the liver [34]. Oral bacteria, particularly periodontopathogens, have been implicated in TMAO metabolism, with evidence suggesting that species like *Porphyromonas gingivalis* and *Fusobacterium nucleatum* may enhance TMA production, thereby contributing to local and systemic TMAO accumulation [8]. Previous in vivo studies have demonstrated that exposure to P. gingivalis can increase circulating TMAO levels and promote endothelial dysfunction, further reinforcing the link between oral dysbiosis, TMAO production, and systemic inflammation [15]. The significant correlation observed between plaque index (PI) and salivary TMAO in our study indirectly supports the notion that bacterial load influences TMAO metabolism, highlighting the need for future microbiome-integrated analyses.

In addition to its potential microbial origins, TMAO is recognized for its pro-inflammatory properties. Our study found a significant positive correlation between TMAO and TNF-α, a key cytokine involved in periodontal and systemic inflammation [33, 35–39]. TNF-α plays a crucial role in periodontal destruction by promoting osteoclastogenesis, matrix metalloproteinase activation, and soft tissue degradation [40]. Elevated TNF-α levels in saliva and serum have been consistently reported in patients with periodontitis [41, 42], particularly in stage III and IV disease [43]. Experimental studies suggest that TMAO may directly stimulate TNF-α production via NF-κB activation [38, 39], contributing to an amplified inflammatory response. These findings suggest that TMAO may not merely be a metabolic byproduct but an active participant in the inflammatory cascade, potentially influencing disease progression.

After evaluating differences in TMAO and TNF-α levels between groups, we further examined the correlation between salivary and serum levels of these molecules. The strong correlation between salivary and systemic TMAO concentrations reinforces the potential of salivary diagnostics as a surrogate for systemic inflammatory status. While serum TMAO has been widely associated with cardiovascular disease risk [15], with meta-analyses reporting its predictive value for atherosclerosis and major adverse cardiovascular events [44], our study specifically excluded individuals with diagnosed cardiovascular disease. The observation that TMAO was elevated in periodontitis patients suggests that periodontal inflammation alone may contribute to increased TMAO production. This aligns with previous reports indicating that TMAO levels can be influenced by chronic inflammatory conditions [38, 39], independent of classical cardiovascular risk factors.

One of the key strengths of this study is its controlled evaluation of salivary and serum TMAO and TNF-α levels, which allowed for the isolation of periodontitis-associated changes from potential systemic confounders. By excluding individuals with known cardiovascular disease and other systemic disorders, we were able to specifically assess the relationship between periodontal inflammation and TMAO metabolism. Additionally, the use of both salivary and serum samples enhances the translational relevance of our findings, supporting the potential application of salivary TMAO as a non-invasive biomarker in periodontal disease monitoring.

However, several limitations should be noted. First, the cross-sectional design of the study limits causal inferences regarding the relationship between TMAO, TNF-α, and periodontal disease progression. Longitudinal studies tracking TMAO levels over time in individuals with periodontitis would help determine whether TMAO serves as a predictive biomarker for disease progression or resolution following periodontal therapy. Second, while our findings suggest a microbial contribution to TMAO levels, we did not directly analyze the composition of the oral microbiome. Given the emerging evidence linking periodontopathogens to TMA metabolism, future research integrating metagenomic and metabolomic approaches could provide deeper insights into the interplay between oral bacteria, host metabolism, and inflammatory pathways.

## Conclusions

This study contributes to the growing body of evidence suggesting that salivary TMAO may serve as a potential biomarker for periodontal inflammation, demonstrating strong correlations between TMAO, TNF-α, and clinical periodontal parameters. Importantly, ordinal regression analyses confirmed that higher salivary TNF-α and TMAO levels were significantly associated with higher odds of periodontitis, even after adjusting for age, reinforcing their relevance as potential indicators of periodontal disease risk. While aMMP-8 has been widely studied as an indicator of periodontal tissue degradation, TMAO appears to provide complementary information, reflecting broader inflammatory pathways that may bridge oral and systemic health. However, given the complexity of TMAO metabolism and its interactions with microbial and host inflammatory responses, further studies are needed to determine its specificity and utility in clinical practice. Future research should focus on longitudinal studies, in vitro and in vivo mechanistic analyses, and microbiome-metabolome integrations to better understand TMAO’s role in periodontitis pathogenesis and its potential application in personalized periodontal diagnostics and therapeutic strategies.

## Data Availability

No datasets were generated or analysed during the current study.

## Footnotes

1 Williams probe, Hu-Friedy, Chicago, IL.

2 Invitrogen Human TNF-α ELISA kit, #BMS223-2HS (Bende MedSystems GmbH, Vienna, Austria).

3 LC–MS/MS, ESI Source, Thermo Scientific Accessmax.

4 3.1.9.2 G*Power; https://www.psychologie.hhu.de/arbeitsgruppen/allgemeine-psychologie-undarbeitspsychologie/gpower.html).

5 SPSS for Windows v.26, IBM SPSS Inc., New York, NY, USA.
